# Multi-View Data Analysis Techniques for Monitoring Smart Building Systems

**DOI:** 10.3390/s21206775

**Published:** 2021-10-12

**Authors:** Vishnu Manasa Devagiri, Veselka Boeva, Shahrooz Abghari, Farhad Basiri, Niklas Lavesson

**Affiliations:** 1 Department of Computer Science, Blekinge Institute of Technology, 371 79 Karlskrona, Sweden; veselka.boeva@bth.se (V.B.); shahrooz.abghari@bth.se (S.A.); 2 iquest AB, Hägersten, 126 26 Stockholm, Sweden; farhad.basiri@iquest.se; 3 Department of Software Engineering, Blekinge Institute of Technology, 371 79 Karlskrona, Sweden; niklas.lavesson@bth.se

**Keywords:** evolutionary clustering, multi-view clustering, multi-instance learning, closed patterns, streaming data, formal concept analysis, smart buildings

## Abstract

In smart buildings, many different systems work in coordination to accomplish their tasks. In this process, the sensors associated with these systems collect large amounts of data generated in a streaming fashion, which is prone to concept drift. Such data are heterogeneous due to the wide range of sensors collecting information about different characteristics of the monitored systems. All these make the monitoring task very challenging. Traditional clustering algorithms are not well equipped to address the mentioned challenges. In this work, we study the use of MV Multi-Instance Clustering algorithm for multi-view analysis and mining of smart building systems’ sensor data. It is demonstrated how this algorithm can be used to perform contextual as well as integrated analysis of the systems. Various scenarios in which the algorithm can be used to analyze the data generated by the systems of a smart building are examined and discussed in this study. In addition, it is also shown how the extracted knowledge can be visualized to detect trends in the systems’ behavior and how it can aid domain experts in the systems’ maintenance. In the experiments conducted, the proposed approach was able to successfully detect the deviating behaviors known to have previously occurred and was also able to identify some new deviations during the monitored period. Based on the results obtained from the experiments, it can be concluded that the proposed algorithm has the ability to be used for monitoring, analysis, and detecting deviating behaviors of the systems in a smart building domain.

## 1. Introduction

The domain of smart buildings is growing rapidly these days. Today’s buildings are equipped with various smart and automated systems such as heating, ventilation, and air conditioning; smart lighting; shading; etc. In order to accomplish the desired functionality, these systems work collectively. Buildings in today’s urban societies generate up to 40% of the total carbon dioxide emissions [1,2]. Along with helping us in our everyday activities, smart systems also play an essential role in energy-saving [1]. The majority of the energy used in these systems is wasted due to operational problems [3]. It therefore becomes appealing to be able to understand, analyze, and monitor the behavior of such systems. Smart buildings are equipped with multiple sensors used to facilitate operations and monitor the systems continuously. These sensors collect a large number of heterogeneous data, which becomes difficult to interpret and analyze. The heterogeneous nature of the data can be linked to a wide range of sources or sensors from which they are collected.

There have been many studies, for example, as shown by the reviews of Farzaneh et al. [1] for a wider area of smart buildings and by Mbiydzenyuy et al. [4] in district heating (DH), that utilize data mining and Machine Learning (ML) to analyze and interpret this large amount of data. Data generated from the sensors have specific characteristics such as heterogeneity, streaming nature, concept drift, etc. that need to be considered during analysis. In addition to the data characteristics stated above, another common hindrance for interpreting real-world data is that they are often not labeled. In order to cope with unlabeled data, unsupervised ML techniques such as clustering analysis can be used. Clustering analysis is the task of grouping data instances based on their similarities into few clusters. Traditional clustering algorithms do not address all the challenges stated, and this calls for a need to develop novel hybrid approaches.

This study demonstrates how the MV Multi-Instance Clustering (MV-MIC) algorithm [5] can be successfully used to analyze and interpret the data in the smart building domain. As the name suggests, the proposed algorithm can address challenges that both multi-view (MV) and stream clustering algorithms can. An MV clustering analysis approach is used to find a consensus clustering solution to group data from across different sources, where each source represents a different perspective or view of the monitored phenomenon. The MV essence of the used stream clustering algorithm helps in addressing challenges such as heterogeneity, streaming nature of sensor data, concept drift phenomenon, and non-labeled data. As stated before, many smart building systems work in coordination, and it is essential to analyze them together. The proposed algorithm can analyze a single system and preform an integrated analysis of two or more systems. Therefore, the flexibility of the algorithm makes it suitable for monitoring different contexts.

The main contributions of this study can be summarized as follows. This study proposes and analyzes different approaches for successful monitoring and analysis of smart building systems. The proposed context-aware approach analyzes and monitors the systems by taking the contextual circumstances into account. In comparison, the other proposed technique, namely integrated system analysis, allows monitoring and analyzing a system in integration with other systems, which is very useful in a domain such as smart buildings where different systems work in coordination. The benefits of using visual data mining are also highlighted. Different visualization techniques for easy interpretation of results generated at each stage of the algorithm are proposed. These visualizations help domain experts to understand the results, monitor any changes in the system, identify the correlations between different views, and detect system failures. Based on the results obtained from the experiments, it can be stated that the proposed approach can be used for continuous monitoring and analysis of the systems in the smart building domain.

The rest of the paper is organized as follows. Section 2 gives an overview of the concepts and methods used in the study. It is followed by Section 3, which presents a brief overview of the related research. Section 4 describes the data used and an MV data analysis approach and introduces visualization techniques proposed in the article. This is followed by Section 5, which provides information about data preparation, experiments, and results. Finally, Section 6 presents the applicability and limitations of the study, and Section 7 is devoted to the conclusion and future work.

## 2. Background

This section introduces and presents a brief overview of various concepts used in the study and the areas related to them. Section 2.1 and Section 2.2 describe clustering algorithms under which the proposed algorithm can be classified. In Section 2.3, Multi-Instance learning, a type of learning technique that is used in the study, is introduced. This is followed by providing an introduction to distance measures in Section 2.4, used to measure how close two clustering solutions are. Finally, formal concept analysis and closed patterns, which are used in the final phase of the algorithm, are described in Section 2.5 and Section 2.6 respectively.

### 2.1. Multi-View Clustering

In MV data, a single data point can be represented in different perspectives or views [6]. Such data are generally acquired from a wide range of sources, are heterogeneous, and usually complement each other. For example, an image and a text describing the same situation can be considered as its two views. Alternatively, a single operation of a system in a smart building domain can be analyzed using different perspectives such as contextual conditions, performance indicators, and operational characteristics. MV clustering is a technique in which the complementary knowledge from different views is extracted, and a model representing all the views is obtained [7].

### 2.2. Stream Clustering Algorithms

Clustering techniques have been traditionally used to categorize data with similar characteristics into a group. Data points belonging to the same cluster are identical to each other and different from those grouped into other clusters.

Stream clustering algorithms belong to a sub-branch of clustering algorithms that deal with streaming data. Stream refers to infinite, non-stationary data that are continuously generated. As the data generation occurs at a high pace, it is impossible to do random access or store all the incoming data [8]. Streaming data are generally not labeled, and hence clustering is one of the most suitable learning techniques for it [9]. Due to the nature and volume of the data generated, stream clustering algorithms should be capable of performing the task considering the memory and time constrains. Apart from these, concept drift is one of the main challenges to be addressed in data stream clustering [9], a phenomenon where the data characteristics tend to change over time. A stream clustering algorithm should be able to adapt to these changes for better results. According to Wadewale et al. [10], drifts can be categorized into six different types, namely sudden, incremental, gradual, recurring, blip, or noise.

### 2.3. Multi-Instance Learning

Multi-Instance (MI) learning is a learning technique in which each data object is a bag consisting of a set of data instances, unlike the traditional learning techniques, where a data object is one data instance [11]. In supervised MI learning, the whole bag is labeled. For example, a picture consisting of both water and sand is labeled as a beach. MI learning has various applications ranging from image classification based on the content [12] to the diagnosis of a disease based on images [13].

#### Multi-Instance Clustering

MI clustering is considered as an unsupervised MI learning, where the data objects are unlabelled bags of instances. Two main advantages of MI clustering over supervised MI learning are that (1) data obtained in many real-world scenarios are not labeled, and it is generally costly to obtain the labels for bags; (2) just like traditional clustering, it is capable of detecting the inherent structure of the data [14]. Even though MI clustering has some similarities with the general clustering algorithms, it cannot be viewed entirely like them. Unlike traditional algorithms, where a single instance is considered as a data object, MI clustering considers a bag of instances. As different instances might show distinct functionalities, it is essential to take into consideration the behavior and relationships of the instances in the bags while grouping the bags into clusters [14].

### 2.4. Distance Measures

In clustering analysis, different types of distance measures are applied during the modeling phase to find the similarity between the instances. Some of these, such as Euclidean and Manhattan distances, are commonly used for traditional single instance clustering methods. These metrics are not suitable for MI learning as the instances are handled as a set of bags. A new distance metric, Hausdorff distance, is proven to be more efficient in these scenarios [14,15,16].

#### Hausdorff Distance

Maximal Hausdorff distance [15], minimal Hausdorff distance [16], and average Hausdorff distance [14] are different kinds of Hausdorff distances presented in the literature. Maximal Hausdorff distance is initially used to measure the distance between two bags in [15] and later on applied to MI learning. Zhang et al. [14] propose average Hausdorff distance, as the other two metrics have not proved to work well in most MI learning problems. Outliers can affect the maximum Hausdorff distance, while minimal Hausdorff distance may be sensitive to the distance between the nearest pair of instances in two bags [14]. Therefore, average Hausdorff distance is considered in the study. Average Hausdorff distance can be calculated using the following formula:

(1) $$H\left(I,J\right)=\frac{\sum_{i\in I}\min_{j\in J}dist\left(i,j\right)+\sum_{j\in J}\min_{i\in I}dist\left(i,j\right)}{|I|+|J|}.$$

In the above equation, *I* and *J* represent bags of instances, and dist(i,j) is the Euclidean distance between instance *i* from bag *I* and instance *j* from bag *J*.

### 2.5. Formal Concept Analysis

Formal Concept Analysis (FCA) [17] is a process for extracting concept hierarchy from a set of objects described by their properties. FCA supplies the user with means for building and visualizing the concept hierarchies, which groups objects and properties concurrently. Hence, it can be considered a conceptual clustering method and is used in various fields of data mining, information retrieval, and knowledge discovery.

FCA builds a *formal context* and derives a *concept lattice* from it. The formal context is a table where the rows correspond to the set of objects *O* and the columns correspond to the set properties or attributes *A* that the objects can possess. If an object possesses a property, then it is represented by a cross in the table. It is a binary relation defined on the Cartesian product O×A.

The *concept lattice* is a hierarchical structure composed of formal concepts. Each concept is a pair of objects and the properties shared by them. A concept can be represented using a pair (X,Y), where *X* is a subset of objects and *Y* is a subset of attributes (properties); the objects in the concept share these properties and vice versa. In the concept lattice hierarchy, for each concept, there exists a super-concept and sub-concept. Concepts (https://pypi.org/project/concepts/0.9.1/, accessed on 30 July 2021), a python module containing the implementation of FCA, is used.

### 2.6. Closed Patterns

Given a sequence database, sequential pattern mining is defined as the problem of finding regularly reoccurring ordered patterns [18]. A pattern is frequent if it has a support greater than or equal to the chosen support threshold. For a sequential database T and pattern *P*, support of *P* is the number of times *P* occurs in T. In larger databases, there might be many frequent patterns that can be difficult to analyze. In such cases, it is advantageous to use closed patterns. A pattern *P* is said to be a closed pattern if it satisfies the following two criteria:

1. It is a frequent pattern.
2. There is no super pattern with the same support as *P*.

BIDE [19], a famous frequent closed sequential pattern mining algorithm, is used to extract patterns. The python module prefixspan (https://pypi.org/project/prefixspan/, accessed on 30 July 2021), which has the BIDE implemented, is used in the study.

## 3. Related Work

There are many recent studies in the field of MV clustering. These include surveys [6,20,21] that summarize the work done or articles proposing novel algorithms [7,22,23,24,25] to address the challenges in the field. While Fu et al. [6] have compared the performance of the selected MV clustering algorithms on real-world data sets, Yang et al. [21] and Chao et al. [20] have categorized the MV clustering algorithms into different categories. In [21], categorization is based on principles and mechanisms used in the algorithms, whereas in [20], clustering algorithms are grouped into either generative or discriminative clustering. In addition, in [20], the authors have also connected MV clustering to other related areas and listed out potential open problems in the area. Huang et al. [7] in their recent study propose a novel MV clustering algorithm based on co-clustering and bipartite graphs. The authors of [22,23] have proposed algorithms that are capable of handling incomplete or missing data in the views. Jiang et al. [24] in their work have considered MV clustering as a multi-objective optimization problem and compared how five multi-objective evolutionary algorithms work for the considered problem. In cite [25], non-negative matrix factorization is used to cluster data across the views.

Compared to the field of MV clustering, MV stream clustering is still in its early stages [26,27]. However, it is gaining more prominence due to large amounts of streaming data being generated across diverse fields. MV stream clustering algorithms are designed to address streamed and multi-viewed data challenges, such as data generation at a high pace and volume, heterogeneity, and concept drift. The authors of [26,27] propose novel algorithms to address the challenges in the field. In [27], the authors use non-negative matrix factorization for incomplete data sets, whereas [26] propose an algorithm based on support vectors.

Artificial intelligence methods, especially ML techniques, are used in the smart building domains for various monitoring, analysis, prediction, and outlier detection tasks to achieve the desired final outcome in terms of energy efficiency, cost reduction, etc. Jafari-Marandi et al. [2] in their work propose a self-organizing map clustering algorithm, distributed decision model, and a homogeneity index used to evaluate the clusters. The algorithm clusters buildings based on their energy profiles. Such clustering can help reduce primary energy consumption. They use the distributed decision model for operational decisions on the building clusters.

Several studies are focused on the DH network, where the produced heat from the primary side is used for heating and supplying domestic hot water to buildings connected to the network [4,28,29]. For example, Mbiydzenyuy et al. [4] review the current state-of-the-art works related to the use of ML in the DH domain. The authors highlight the need for ML to plan, monitor, optimize, and control these systems. They have also listed some goals that needs to be accomplished to increase the impact of ML in the DH domain. Barriers that can hinder the achievement of these goals and ways to overcome them are also stated. Abghari et al. [28] propose an MV clustering approach to identify sub-optimal behaviors of DH substations by considering their geographical locations. The proposed method offers two different analyses, namely step-wise and parallel-wise MV clustering. The step-wise analysis performs a hierarchical clustering based on different feature sets considered in each view. Whereas in the parallel-wise analysis, clustering solutions built upon two views are compared to determine the similarities and variations. Theusch et al. [29] present an ML pipeline using clustering and regression analyses for monitoring and fault detection in DH substations using smart meter data. The authors also identify two key performance indicators, primary return temperature, and the difference of primary supply and return temperatures.

Eghbalian et al. [30] study the heating system, which is part of a Heating, Ventilation, Air Conditioning, and Refrigeration (HVAC&R) system in a smart building domain. They propose an MV data analysis method for monitoring the smart control valve system, which is a part of the heating system. The proposed approach was able to detect deviating behavior successfully. Shchetinin [31] uses clustering to be able to forecast electricity consumption by building consumer profiles. Smart meter data are used to build the profiles.

There have not been many works using MV stream clustering to analyze and/or monitor system behavior in the domain of smart buildings. This work attempts to bridge this gap.

## 4. Materials and Methods

This section describes data analysis, visualization, and pattern mining methods proposed in this article. Section 4.1 provides information about the data used for evaluation purposes. The remaining two subsections are devoted to advanced methods for MV integration analysis and continuous pattern mining of smart systems sensor data. Section 4.2 discusses a method of context-aware modeling of system behavior and integration analysis of its performance, while Section 4.3 deals with the visualization and pattern mining.

### 4.1. Data

In this study, real-world sensor data are used to evaluate the performance of the MV-MIC algorithm, [5] in the field of smart buildings. Data used are obtained from a company based in Stockholm, Sweden. In the smart buildings domain, many systems work together. One of these systems, HVAC&R, is considered in the study. The primary focus is on the heating and tap-water sub-systems. The data used in this study belong to a health-care building located in Stockholm, Sweden. It is a three-storey building which has approximately 2100 m${}^{2}$ floor area. More details about the sensors from which the data are collected and the features included in the study can be found in Section 5.2, where the experimental scenarios are discussed.

### 4.2. Multi-View Data Analysis Approach

The work of smart systems is often monitored by multiple sensors, each capturing a different factor of operational or contextual circumstances, due to which the data have a heterogeneous nature and provide different perspectives (views) about the studied system. Modeling the behavior and analyzing the performance of such smart systems are often very complex and can be computationally demanding. All these make the monitoring task very challenging, requiring ML and data mining models that are not only able to continuously integrate and analyze MV streaming data, but also are capable of adapting to concept drift scenarios of newly arriving data.

#### 4.2.1. Context-Aware Modeling of System Behavior

MV Multi-Instance Clustering is an MV stream clustering algorithm that is proposed in [5]. The MV-MIC algorithm can simultaneously monitor the local clustering models in each view and build an integrated global model, which can be used to find the correlations between the views. The proposed algorithm can continuously monitor and analyze the streaming data. When a new data chunk arrives, the clustering models in each view are updated using Bi-correlation MI clustering. This is followed by building a global model that consists of formal context and a formal concept lattice. MV-MIC takes advantage of extracting closed patterns to obtain the most frequent correlations between the views. The global model built by using FCA helps in analyzing and comparing the correlations between different views of consecutive data chunks.

In this section, we consider and demonstrate how the MV-MIC can be used for context-aware modeling and analysis of smart building system behavior and performance. Each system of the smart building can be monitored from different perspectives or views. Sensors are used to collect a large volume of valuable data about the system operation, context, and performance. For example, different contextual factors such as outdoor temperature, the social behavior of people, etc., can influence the system’s operating modes and performance. Evidently, to get a realistic evaluation of the system’s behavior, its performance should be assessed by analyzing its operation under different contextual factors, i.e., different perspectives should be studied and linked. Such a division of the system’s characteristics will facilitate the domain experts in better understanding how the system’s performance correlates with its operating modes and is affected by different contextual circumstances.

Let us consider a streaming scenario where data are analyzed in chunks. That is, each data chunk *t* contains *N* most relevant data characteristics of the monitored system. These characteristics (attributes) are selected via a preliminary discussion with domain experts. Hence, the data chunk corpus consists of *N* different data sets, one per monitored characteristic, and each data set contains $n_{t}$ daily time-series profiles (measurements). Note that the data chunks can have different sizes; i.e., they can contain different numbers of daily profiles. The available data attributes are further analyzed together with the domain experts and are separated into different views with respect to the information they provide about the monitored system. For example, some parameters may be related to the system operating behavior (operational parameters); others can present different kinds of contextual factors or define system performance indicators. Note that in our description hereafter, system’s operational, contextual, and performance characteristics are denoted by views 1, 2, and 3, respectively.

**Figure 1:**
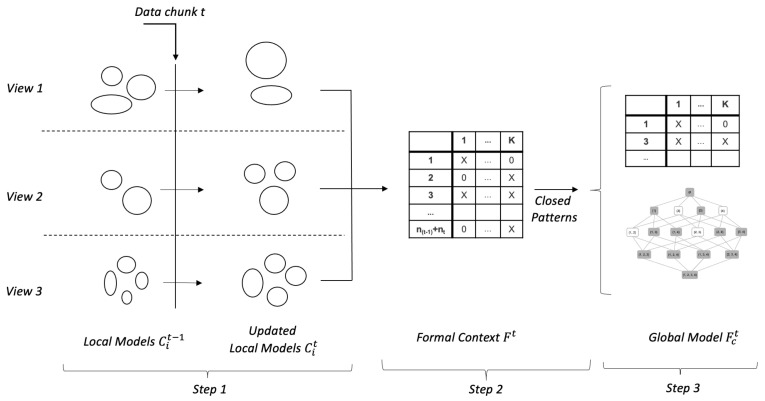
A schematic illustration of different steps of the MV-MIC algorithm.

At each newly arrived data chunk *t*, the approach performs three distinctive steps as illustrated in Figure 1:

Step 1: Update local clustering models
  (a) Initially, for each view *i* (i=1,2,3), integrated daily profiles are created by using the view attributes ($N_{i}$ in total); i.e., each integrated daily profile is a $N_{i}$-dimensional vector that consists of the aggregated values of the features (attributes) of view *i* (i=1,2,3).
  (b) A local clustering model $C_{i}^{t}$ (i=1,2,3) is produced at each data view.
  (c) A clustering model $C_{i}^{t-1}$ (i=1,2,3) built at previous data chunk t−1 is updated by the corresponding model ($C_{i}^{t}$) produced on the new data chunk by applying the Bi-correlation MI clustering algorithm [5].
Step 2: Build a formal context
  A formal context matrix $F^{t}$, which consists of all MV patterns supported by the updated clustering solutions, is built. Each row *j* ($j=1,2,\ldots ,(n_{t-1}+n_{t})$) of $F^{t}$ is a *K*-length binary vector, where $K=k_{1}+k_{2}+k_{3}$, and $k_{i}$ (i=1,2,3) is the number of cluster labels in the updated clustering solution of view *i*.
Step 3: Generate a global model
  (a) Closed (most frequent) patterns, denoted by $F_{c}^{t}$, are extracted from $F^{t}$. These patterns present most typical current correlations among the three views, i.e., those that are supported by the chunks t−1 and *t*.
  (b) The set of the closed patterns $F_{t}^{c}$ is used to generate a concept lattice that describes the hierarchical organization of the identified concepts.

The fourth step of the proposed MV analysis approach can be considered as the post-analysis that can be conducted on the results produced by its application.

Step 4: Analysis
  The produced global models can be used to study and analyze the system behavior and performance, e.g., by conducting some of the following:
  (a) Analysis of system behavior:
    The extracted closed patterns ($F_{c}^{t}$) can be studied to understand the current behavior and performance of the system.
  (b) Identifying deviating behavior:
    i. The patterns belonging to $F_{c}^{t}$ can be benchmarked to those available in $F_{c}^{t-1}$ (i.e., the most typical patterns identified at the previous data chunk t−1) to discover deviating or unseen behavioral modes.
    ii. The hierarchical relationships revealed by the concept lattices built on $F_{c}^{t}$ and $F_{c}^{t-1}$ can also be studied for gaining additional insight into the temporal behavior of the system.
  (c) Tracking back system behavior:
    The system behavior can be studied and tracked back for a longer period than two consecutive chunks by analyzing, e.g., the sets of extracted closed patterns $\left\{F_{c}^{p}|p=t,t-1,\ldots ,t-q\right\}$ (q≥2) covering the studied period.

#### 4.2.2. Integration Analysis of System Performance

A smart building uses various technologies to optimize the building’s performance and energy efficiency by sharing information about what goes on in the building between systems. This information is used to automate various processes, from HVAC&R to lighting and security. The most fundamental feature of a smart building is the interconnections of its core systems.

In this section, we propose and discuss the extension of the MV-MIC approach to conduct integration analysis of few different systems that together realize the functionalities of a smart building system. The ability to conduct integrated analysis benefits the understanding of the correlations between different views of the systems involved and how these affect the performance or behavior of a larger system (for ex: HVAC&R) in consideration.

As stated, a smart building has various systems that work both individually and in integration with other systems. Each system can further have multiple sub-systems performing their own designated task or collaborate with other systems. For example, in the HVAC&R, which is responsible for heating, including hot water system, ventilation, air conditioning, and refrigeration, a designated sub-system is responsible for each of these. The contextual conditions such as outdoor temperature and inhabitant behavior are the same for all these systems, influencing their performance. Note that more number of inhabitants yields increased hot water consumption and demands for higher ventilation. As an illustration of how the systems work in coordination with the selected HVAC&R system, let us consider the operation of the hot tap-water system (hear after referred to as tap-water system), which is linked to the heating system. Hot water returned from the heating system is transferred to the tap-water system through a heat exchanger and used to heat the tap water. Suppose this temperature received from the heat exchanger is insufficient. In that case, the tap-water system opens one of its valves to release some more hot water to obtain the desired temperature. Such interlinks can be seen among other systems as well. As the systems work in coordination with each other, a better understanding of the systems’ operation can be obtained by applying flexible data analytic algorithms capable of conducting integrated MV analysis of multiple systems. This integrated analysis can help to identify behaviors that are challenging to detect when the systems are analyzed individually.

**Figure 2:**
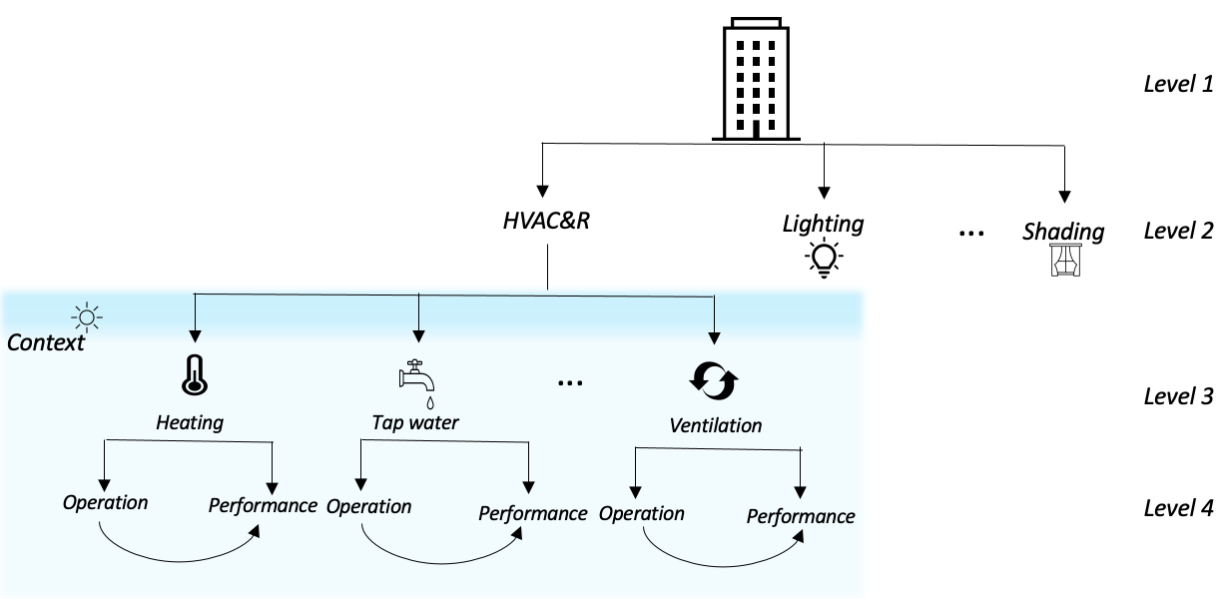
Example of integrated systems in a smart building.

The proposed MV-MIC algorithm perfectly fits into this role of an integrated system analysis tool. It can be used to analyze each sub-system individually or in combination with sub-systems with respect to different perspectives or views such as operating modes, context, and performance. Figure 2 shows a high-level overview of how the systems work in integration in a smart building. It can be seen that the systems of a smart building can be represented using a hierarchical structure. After the local models in each view are updated, one can dynamically decide and select views to be analyzed further and find correlations between them. Then, the global model is built using these chosen views. Note that Figure 2 is included only to illustrate how the systems of a smart building can be grouped together in a hierarchical structure. Experiments in the current study are only conducted on the heating and tap-water systems shown in the figure.

Assume that at data chunk *t*, the operation and performance of two linked systems, e.g., heating and tap-water (or heating and ventilation), are studied. These systems work under the same exogenous circumstances. In this context, a few different scenarios can be analyzed to understand and gain better insight into the systems’ integrated behavior:

A. Meta-integration analysis of two systems:
  The MV-MIC algorithm, described in Section 4.2.1, can be applied individually to each system’s data. As a result, two sets of closed patterns linking the three views’ of each system are extracted. These can be separately analyzed for each system to understand the system behavior. In addition, the two sets of extracted closed patterns can be aligned to each other with respect to the features in the contextual view in order to identify some interconnections between the systems affecting and explaining their performance.
B. Inter-study integration analysis of two systems:
  The two systems’ data views can be considered together, and the MV-MIC algorithm can be used to analyze the systems. In this scenario, the extracted closed patterns will represent integrated systems’ profiles linking operating modes and performance of the two systems under the monitored contextual conditions.
C. Integration analysis of selected views:
  Selected views from the two systems can be analyzed together, e.g., only performance indicators of heating and tap-water may be considered. Such analysis may reveal, e.g., how the performance of the tap-water system is correlated to the heating system.

There are various advantages of using integration analysis. One can initially analyze the sub-systems and then integrate them to obtain a full overview of the whole system. It will enable us to utilize the benefits provided by the MV clustering completely. Considering sub-systems helps in identifying hidden cluster groups. Note that there might be difficulty identifying proper clusters when the complete system is studied due to the increased complexity of having many different types of heterogeneous features.

The proposed approach is capable of performing both horizontal and vertical integration of the knowledge obtained through the analysis of the systems. Vertical integration of the knowledge can be done when a new data chunk arrives; Bi-correlation MI clustering is used to update the existing local clustering solutions based on the newly arriving data. Horizontal integration can be done between different views and sub-systems as per the requirement, e.g., see integration scenarios B and C above.

### 4.3. Data Visualization and Analysis

This section presents how the MV-MIC can be combined with different data visualization techniques for facilitating continuous monitoring, analysis, and pattern mining of smart building system behavior and performance. We demonstrate how different visualization techniques could be used to better understand and monitor the system performance and operation. The proposed new approach of continuous visual tracking for each data chunk helps to quickly identify and track the changes over time.

The amount of data generated by the smart building systems is enormous and difficult to interpret; this can be addressed using visual data mining [32]. Visualization can be considered as a part of data mining and knowledge discovery [33]. It helps in representation and eases the task of understanding a huge volume of data that are difficult to present and analyze in a textual form [32,34]. One of the significant advantages of visual exploration, as stated by Keim [34] is that it is capable of handling heterogeneous and noisy data better than what statistical or ML techniques have to offer. The paper proposes a visual data mining based approach to detect abnormal behavior based on historical operational data.

In the current study, visualization is used to represent the outcomes of different stages of the proposed algorithm. These visualizations can be used by domain experts to better understand the operation and performance of the system.

#### 4.3.1. Visualization of Results

As stated before, visualization increases the understandability of complex results. In [32], the authors highlight the need for continuous visualization in a data mining process. Inspired by this, we propose suitable visualizations of the results obtained in each phase of the MV-MIC algorithm. This visualization facilitates the domain expert in analyzing and better understanding the system behavior and performance by presenting details that are not visible in the end results.

1. Visualization of local models:
  Each local clustering model produced at Step 1 of the algorithm captures information about the most typical working/performing modes or conditions of the studied system in the respective view. In order to facilitate the perception of the information summarized in each local clustering model $C_{i}^{t}$ (i=1,2,3), it can be visualized by a $k_{i}\times N_{i}$ matrix that contains average values of the corresponding view’s attributes w.r.t. the identified typical working scenarios (clusters). The matrix can be colored by using different color intensities for each row by taking into account the size of the cluster it presents; e.g., see Table 3. Such colored matrices can be used to visually inspect the system’s working scenarios in the different perspectives considered. This can further facilitate the detection of deviating/underperforming scenarios by comparing the local models built on two consecutive data chunks of the system. The data presented in each matrix can also be sorted by a selected attribute, which can further facilitate the analysis and understanding of the system behavior. For example, Tables 3 and 8 can be ordered w.r.t. PHL and then aligned to each other.
2. Visualization of formal context:
  A formal context $F^{t}$ is built at Step 2 of the algorithm using the data points of the current and previous chunks. Each data point of $F^{t}$ can be represented (labeled) by a three-length vector (string) that contains the respective labels from the three views’ local models. This can be used to visualize an extract of *m* data points using an m×3 matrix (3 represents the number of views). Each column of this matrix can be colored by the respective view color used in the visualization of the view’s local model. In addition, each cell may have different color intensity, similar to the local model matrices. Such visualization can be used to study a specific period of the system work by allowing them to conduct higher-order comparison and analysis of the system’s daily behavior and performance under the different contextual conditions in the studied period. For example, two tables presenting, respectively, two consecutive weeks of the system work can be visualized and studied by request, e.g., see the two tables in Table 6. These can reveal, e.g., that the system has moved in a different operating mode in the second studied period (week), although the contextual conditions and performance measures have not changed from the first week. This may be an indication of a problem and can be further studied by the domain experts. Note that the above tables can also be sorted w.r.t. the labels in a column by choice, which can further facilitate the comparison.
3. Visualization of concepts linking three views:
  A set of closed patterns $F_{c}^{t}$ is extracted from $F^{t}$ at Step 3 of the proposed approach. This set of closed patterns is used to build a concept lattice (global model) at chunk *t*. The concepts linking three views can be presented in a table similar to the one used for local clustering models, but containing average values of the attributes from all three views; e.g., see Table 7. In addition to this table, a tripartite graph can be created to visualize the correlations between the three views’ clusters revealed by those concepts. A tripartite graph $G_{t}=\left(V_{1}^{t},V_{2}^{t},V_{3}^{t},E_{12}^{t},E_{23}^{t}\right)$ can be created for each data chunk *t*, where the first three components ($V_{1}^{t},V_{2}^{t}$ and $V_{3}^{t}$) are, respectively, the three vertex sets of the graph and the remaining two ($E_{12}^{t}$ and $E_{23}^{t}$) are the edge sets. Note that $V_{i}^{t}$ (i=1,2,3) is the set of cluster labels of clustering solution $C_{i}^{t}$ (i=1,2,3). Furthermore, $E_{12}^{t}\subset V_{1}^{t}\times V_{2}^{t}$ and $E_{23}^{t}\subset V_{2}^{t}\times V_{3}^{t}$ present, respectively, the links between local models $C_{1}^{t}$ and $C_{2}^{t}$ and between $C_{2}^{t}$ and $C_{3}^{t}$, which are revealed by the formal concepts of the global model. These edges, when considered together, also present the links between the local models of all three views. For example, see Figure 4: the edge connecting nodes $A_{O0},A_{C0}$, and $A_{P0}$ in all three views. i.e., $V_{1}^{t}$, $V_{2}^{t}$, and $V_{3}^{t}$ represent the correlation between these three clusters from these views. The graph edges can have a different thickness that reflects the size of the concept they present. Furthermore, the nodes in the vertex sets can be colored by using the same color visualization idea as the one applied for the local models. Such a graph visualization can facilitate the domain experts in getting an overall (at a higher level) understanding of the system behavior and performance w.r.t. different contextual scenarios. The comparison of two graphs produced on the data of two consecutive chunks (e.g., $G_{t-1}$ and $G_{t}$) can provide information about newly appeared correlations among the three main characteristics of the system.
  In addition to the table and tripartite graph, each concept can be visualized by plotting its performance mode feature values in a spider chart and further labeling the chart by selected parameters from the other two views (context and/or operation). Such plots will facilitate the visual comparison of the different concepts, e.g., on domain expert request, all heating season concepts can be plotted and inspected.
4. Visualization of concept lattice:
  The set of closed patterns $F_{c}^{t}$ can be used at Step 3 of the proposed approach to generate a concept lattice. The latter can present a complex hierarchical structure. Therefore, it is not considered very useful to visualize the whole lattice. If needed, a sub-lattice can be visualized to illustrate further, e.g., the links of specific three-view concepts with two-view concepts.

## 5. Experimentation and Analysis

This section presents the experimental scenarios investigated in this study along with the results obtained, followed by a discussion. Section 5.1 presents the data pre-processing steps. Section 5.2 describes the used experimental setup and discusses the obtained results. Experimentation is done by considering the heating and tap-water systems independently and together in an integrated scenarios.

### 5.1. Data Preparation

This section describes the data pre-processing steps used before the proposed MV-MIC algorithm is applied. Each of these steps is applied to the newly arriving data chunks.

#### 5.1.1. Outlier Removal

Real-world data often contain data points with a deviating behavior known as outliers or noise. Building a model with such data might negatively impact the performance of the model. Sudden spikes or drops in the measurements can be smoothened using data smoothing techniques belonging to the Median Absolute Deviation (MAD) family. In this study, a Hampel filter [35] is used to replace such outliers with a local median of a sliding window of size *k* (7 in this case). Python module for Hampel filter (https://github.com/MichaelisTrofficus/hampel_filter, accessed on 5 July 2021) is used.

#### 5.1.2. Data Cleaning

Some features in the data have missing values. In the heating or tap-water systems, the values of each feature depends on various factors such as the current outdoor temperature, building occupancy, ventilation, and tap-water usage, to name a few, making it difficult to estimate the missing values correctly. Hence, the rows with missing values are removed.

#### 5.1.3. Data Division

Data over a period of two years (1 January 2019 to 31 December 2020) are used to conduct the experiments. The streaming data set is divided into three chunks. One year of data, that is, from 1 January 2019 until 31 December 2019 are considered as chunk 1. The second-year data are divided into two chunks, i.e., 1 January 2020 until 30 of June 2020 is considered as chunk 2 and 1 July 2020 until 31 of December 2020 is considered as chunk 3. Daily profiles are used in the study as the hourly data are sparse for some features.

#### 5.1.4. Standardization

Each data chunk is standardized using *z*-score. Standardization of the features is done by subtracting the mean value of the feature from each sample and dividing it by the standard deviation of the feature. Equation (2) is used to calculate the *z*-score, where *x* is the sample, *u* is the mean, and *s* is the standard deviation. StandardScaler from the preprocessing module of python Scikit-learn [36] library is used to perform the standardization.

(2) $$z=\frac{x-u}{s}.$$

#### 5.1.5. Estimation of the Number of Clusters

In this study, *k*-means is used to initially cluster the data points in different views. This partitioning algorithm requires *k*, the number of clusters to be known in advance. Therefore, we take advantage of the Silhouette Index cluster validation method for identifying the optimal number of clusters *k*.

The Silhouette Index (SI) for clustering solution *C* of *n* objects is defined as:

(3) $$s\left(C\right)=\frac{1}{n}\sum_{i=1}^{n}\frac{\left(b_{i}-a_{i}\right)}{\max\{a_{i},b_{i}\}},$$

where $a_{i}$ represents the average distance of item *i* from all the other items in the cluster to which the item *i* is assigned, and $b_{i}$ represents the minimum of the average distances of item *i* from items of the other clusters.

### 5.2. Experimental Setup and Results

**Figure 3:**
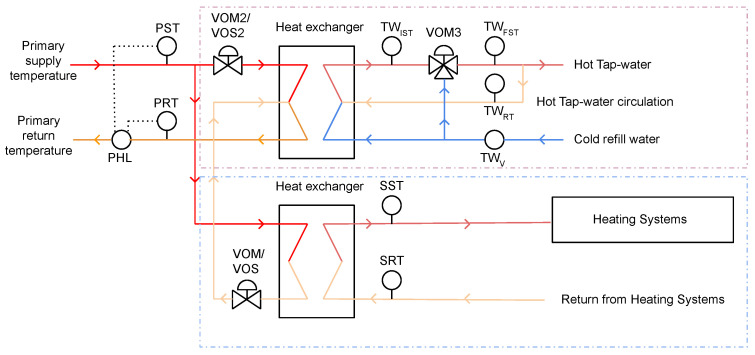
HVAC&R system schematics: dashed purple rectangle represents the tap-water sub-system, and dashed blue rectangle represents heating systems.

The proposed algorithm is suitable for various continuous data mining tasks such as monitoring and pattern extraction at multiple levels (see Figure 2) of the smart building system. In this section, we demonstrate how the proposed algorithm could be used for an MV data analysis of independent systems, scenario A, (Section 5.2.1), as well as for an integrated system, scenario B, (Section 5.2.2) using different experimental settings. More specifically, data from heating and tap-water sub-systems that are part of the HVAC&R system are used for different experiments conducted in the study. Figure 3 illustrates the HVAC&R system schematics, including the tap-water (dashed purple rectangle) and heating (dashed blue rectangle) sub-systems.

#### 5.2.1. Individual Analysis of Heating and Tap-Water Systems

In this set of experiments, the heating and the tap-water systems are individually analyzed to observe their performance independently. These are used to find correlations within the sub-system that are difficult to identify when analyzing an integrated system.

##### Experiments on the Heating System

This experimental scenario monitors the heating system, which is part of an HVAC&R system. The experiment is designed to help domain experts to better understand how the operational, performance, and contextual characteristics affect each other. The system contains various sensors, which are continuously collecting information. These metrics are classified into three views, representing the operation, performance, and context of the system. Details about the features included in each of these views are presented in Table 1.

The system’s operational parameters include the secondary supply and return temperatures, and primary heat load. The average valve openness and its standard deviation together with sub-station efficiency are considered for measuring the performance. For the contextual parameters, average outdoor temperature, along with its standard deviation, are considered.

The proposed algorithm requires that the data in each chunk are initially clustered. Initial clustering in the operating and performance views, that is, views 1 and 2, is done using *k*-means. The optimal value for *k* is identified using SI. For the contextual view (view 3), initial clustering is performed based on the seasons in a year as proposed by [37]. The data are divided into four clusters, namely winter (December to February), early spring and late summer (March, April, October, and November), late spring and early autumn (May and September), and summer (June to August). Details about the number of initial clusters in each chunk for different views can be seen in Table 2.

Considering the chronological order in which the data arrive, MV-MIC is initially applied on chunks 1 and 2. First, the local models in each view are updated using the Bi-Correlation MI-Clustering, which produced 9,4, and 5 clusters for views 1, 2, and 3, respectively. Overview of each of these clusters from different views can be seen from Table 3, Table 4 and Table 5. As stated in Section 4.3.1, these tables are colored based on the size of the cluster and can be used for visualizing different working or contextual modes in each of the views.

After building a local model for each view, FCA is used to integrate them and build the global model, which contains a formal context and concept lattice. The concept lattice has 69 non-empty concepts. Among these, only 32 concepts connect all three views. As stated in Section 4.3.1, visualization of the formal context can be used to compare the system behavior at higher granularity, e.g., a day. To illustrate this, two weeks of data are considered, where week one represents normal system behavior, while week two contains abnormal and sub-optimal behavior. A gap of one week is given in between to make sure that the normal and abnormal behaviors do not coincide. Table 6 presents two weeks of data, that is, from 1 March 2019 to 7 March 2019 and 15 March 2019 to 21 March 2019, representing the system performance in March-2019. From these tables, it can be observed that during week two, the operating mode is always $A_{O4}$, representing the deviating behavior, and a sudden drop in PHL down to 3.33 kW (see Table 3). In comparison, the operating modes for normal behavior during this time are either $A_{O0}$ with an average PHL equal to 24.16 kW or $A_{O3}$ with an average PHL equal to 44.38 kW (the operating modes identified between 1 March 2019 to 7 March 2019). The performance and contextual modes during these weeks are identical. Such tables can help the domain expert identify the issues (here with the features related to the operating mode) and take timely action.

**Figure 4:**
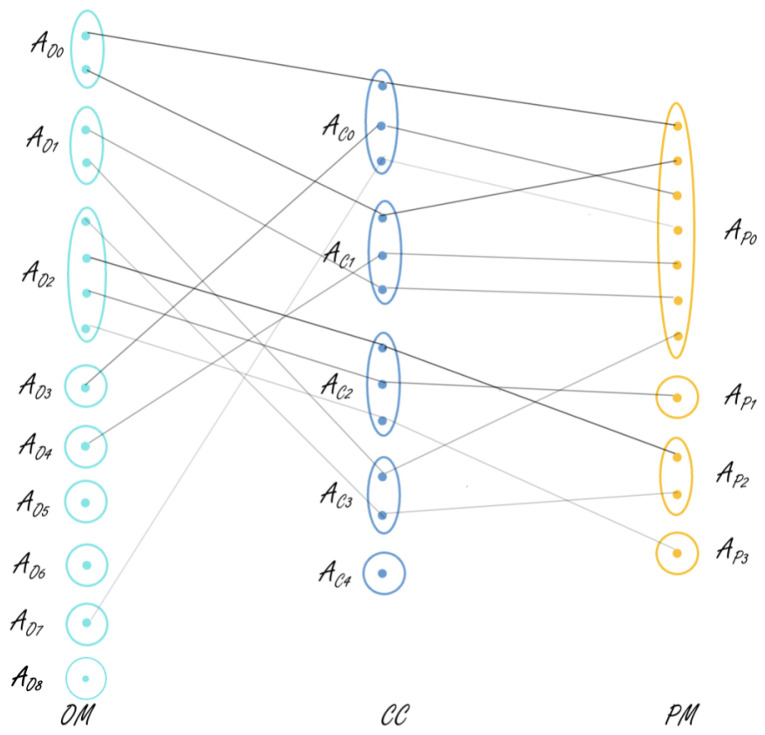
Tripartite graph representing the relationships between different concepts after receiving chunk 2. Color thickness of the edges correlates to the size of the concept.

Next, closed patterns are used to extract the most common or frequent behavioral patterns. Support of ≈2.5% is used in this process, i.e., patterns that cover at least 2.5% of the data are considered to be frequent. There are 513 daily profiles in total when both chunks 1 and 2 are considered. That is, concepts with a frequency of at least 13 are considered. This gave us 31 concepts linking any two views and 11 concepts linking all three views. These 11 concepts are represented in Table 7 and Figure 4. In Table 7, the data are sorted based on the OTM.

From Table 7 it can be observed that there is a sudden drop in the PHL for concept 4, implicating a deviating behavior that matched with the prior information we had regarding issues in the system during March and April 2019. This showcases that the proposed algorithm is capable of detecting abnormal or deviating behaviors. Concepts 9 and 8 are very similar but could have been interpreted as different concepts because of the clustering in view 3, since the months in these concepts belong to different initial clusters.

Figure 4 is a tripartite graph representing all 11 concepts linking three views that are obtained after using closed patterns. This figure showcases the links between all three views and gives the observer an easy understanding of how the views are correlated. The edges of the graph are of varied thickness representing the size of the concept. Concepts with greater size are represented by thicker lines showing a stronger correlation between views, whereas the lighter lines imply that the considered concept is only supported by a few daily profiles, i.e., that the correlation is not strong. For example, the first link in the figure represents a concept linking clusters $A_{O0}$, $A_{C0}$, and $A_{P0}$ in views 1, 2, and 3, respectively. This is supported by a size of 60 daily profiles. The link between clusters $A_{O7}$, $A_{C0}$, and $A_{P0}$ in views 1, 2, and 3, on the other hand, is supported by a concept of size 14. One can also observe from the tripartite graph that some views’ clusters do not take part in any of the three-view concepts, e.g., clusters $A_{O5},A_{O6},A_{O8}$, and $A_{C4}$. These might be involved in two-view concepts. It is interesting to notice that $A_{O5}$ and $A_{C4}$ are the smallest clusters among the others in their local clustering models. $A_{O6}$ and $A_{O8}$ are also of the smallest clusters in view 1.

When chunk 3 arrives, the local clustering models in each view are updated again. The number of clusters in the updated local models are 8, 3, and 6 clusters in views 1, 2, and 3, respectively. These can be viewed in Table 8, Table 9 and Table 10. When comparing the operating modes clusters between Table 3 and Table 8, it can be observed that in Table 3, operating mode $A_{O4}$, which represents deviating behavior, can be considered as an additional mode. All the other modes except $A_{O3}$ from Table 3 can be compared to one of the modes listed in Table 8. That is, clusters $B_{O2}$, $B_{O3}$, and $B_{O5}$ from Table 8 are similar to clusters $A_{O5}$, $A_{O7}$, and $A_{O8}$, respectively, from Table 3. Clusters $B_{O0}$, $B_{O1}$, and $B_{O4}$ in Table 8, on the other hand, are close to clusters $A_{O0}$, $A_{O2}$, and $A_{O1}$, respectively, from Table 3. It can be stated that when the local models are updated, some clusters are retained while some are updated. For the performance modes, the number of clusters are not evenly distributed, and a majority of the instances are grouped into cluster $B_{P0}$ (see Table 9). In view 3, as stated before, the clustering is done based on the seasons of the year, there are some new clusters, some of them are retained while others are updated.

After the global model is built, initially, there are 48 non-empty concepts; among these, 23 concepts connect all the three views. As the number of instances in chunks 2 and 3 combined is 329, support of 8 is used when extracting closed patterns. When closed patterns are used to extract the most frequent patterns, 32 concepts connecting any two views and 14 concepts connecting all the three views are obtained. The latter are represented in Table 11. From this table, deviating behavior is seen in concepts 3,4,9, and 10, where the PHL shows a significant difference from its original pattern. Furthermore, it is interesting to note that all these concepts show deviating behavior with respect to SE. As one can observe, SE in concepts 3, 9, and 10 is negative, while in concepts 4 and 11, it has unexpectedly high (2852%) and low (21%) values, respectively. These results were discussed with the domain expert and it was identified that there were in fact some issues in the system from the end of September till mid-December 2020. This once again showcases the potential of the proposed algorithm in identifying new trends in the data, long-term fault within the system in this scenario.

**Figure 5:**
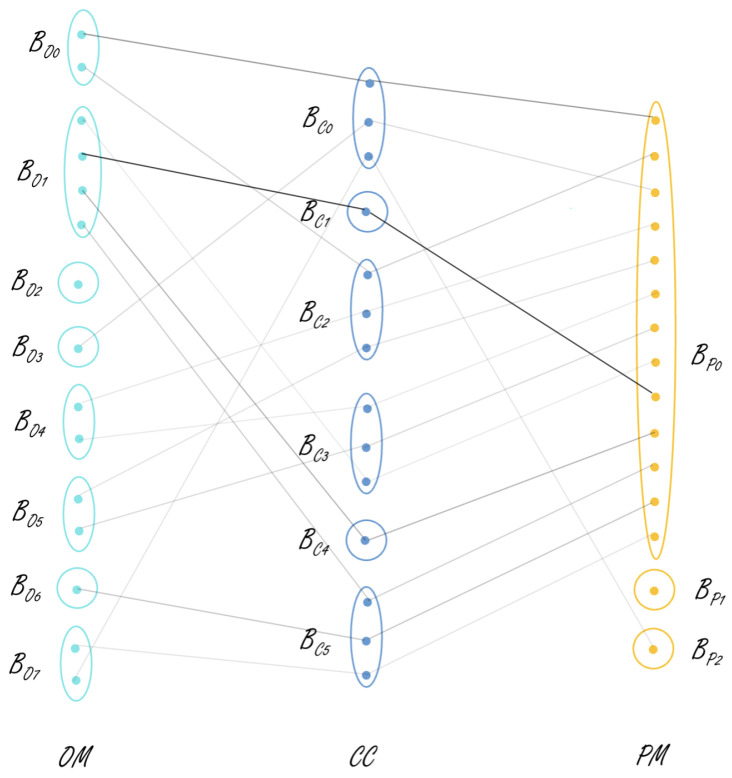
Tripartite graph representing the relationships between different concepts after receiving chunk 3. Color thickness of the edges correlates to the size of the concept.

Figure 5 represents the links between different views of the concepts present in Table 11, which are obtained after using closed patterns. For example, the link between $B_{O1}$, $B_{C1}$, and $B_{P0}$ supported by 80 instances is the strongest correlation and represents concept 13.

**Figure 6:**
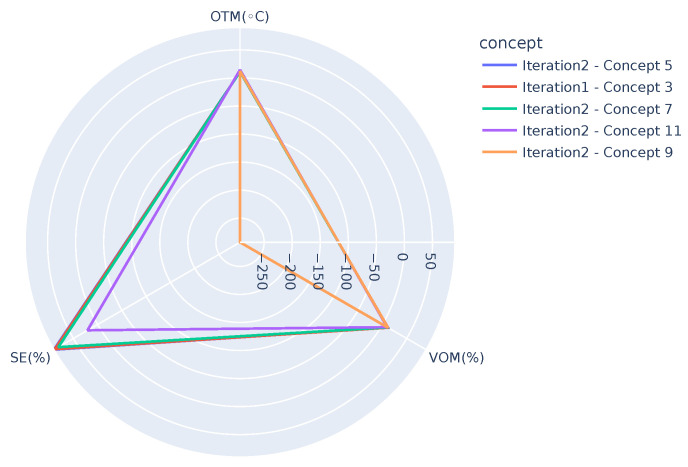
Comparing concepts of both the iterations (after receiving chunk 2 and 3, Iteration 1 and 2, respectively) using the relationship between OTM, VOM, and SE when 10 °C < OTM < 15 °C. Note that some of the concepts in the legend are not visible in the image due to overlap.

**Figure 7:**
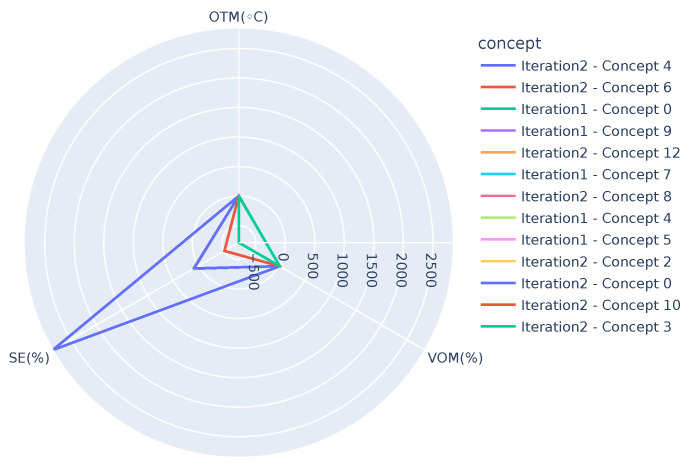
Comparing concepts of both the iterations (after receiving chunk 2 and 3, Iteration 1 and 2, respectively) using the relationship between OTM, VOM, and SE, when OTM < 10 °C. Note that some of the concepts in the legend are not visible in the image due to overlap.

**Figure 8:**
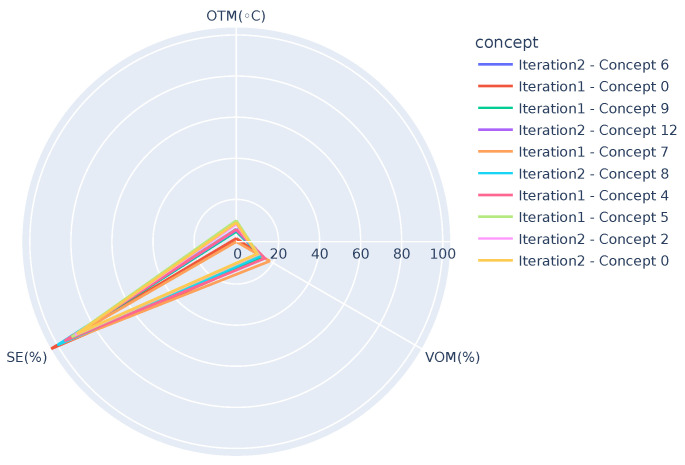
Comparing concepts of both the iterations (after receiving chunk 2 and 3, Iteration 1 and 2, respectively) using the relationship between OTM, VOM, and SE, when OTM < 10 °C and after removing deviating concepts 3, 4, and 10 of iteration 2. Note that some of the concepts in the legend are not visible in the image due to overlap.

In order to get further insight into the differences between the concepts and/or to track any potential drifts, visualization techniques such as the ones shown in Figure 6, Figure 7 and Figure 8 can be used. Figure 6 presents concepts from both the iterations, i.e., one after receiving chunk 2 and the other after receiving chunk 3 when OTM is in the range, 10 °C < OTM < 15 °C. It can be observed that the graphs highlight two extremes, *Iteration2-Concept 9* (SE=−293%) and *Iteration 2-Concept 11* (SE=23%), where the SE (SE ranges from 0 up to 100%. However, due to the generation of hot tap water, it can rise up to 120%.) shows deviation from the other concepts. Similarly, Figure 7 presents concepts when OTM < 10 °C and highlights three deviations, *Iteration 2-Concept 4* (SE=2852%), *Iteration 2-Concept 3* (SE=−789%), and *Iteration 2-Concept 10* (SE=−506%). Note that except for the mentioned concepts, all the others in the figure overlap, showing the similarity among them. Figure 8 presents concepts of both the iterations after removing the deviating concepts when OTM < 10 °C. It can be clearly observed that all concepts are close to one another. As demonstrated, these graphs can be used by domain experts to see the similarities or changes between different concepts, which can help them to identify the changes in the behavior of the system. In the long run, they can also have one such graph for each smaller temperature range (say, for example, 1 or 2 °C). It is expected that concepts in the same temperature range should be similar, so even a small deviation in behavior (gradual concept drift) can also be observed.

##### Experiments on the Tap-Water System

Similar to the experiments conducted for the heating system, individual system analysis is also performed on the tap-water system. Based on the discussion and feedback received from the domain expert, the features characterizing the tap-water behavior are divided into three views, namely operation, performance, and context, as shown in Table 12.

The features measuring the system’s operational parameters include the primary heat load, volume of water used during the day, supply, and return temperatures. For measuring the performance, openness of both the valves used by the tap-water system along with the primary delta (difference between the primary supply and primary return temperatures) are considered. Among the two valves, VOM3, a three-way valve, is responsible for regulating the hot tap-water temperature to be around 60 °C. In order to maintain this temperature, the valve might sometimes allow cold water to be mixed with hot water. Hence, its standard deviation is not considered as it is not varied often. The outdoor temperature and the openness of the valve from the heating system are considered as the contextual parameters. The valve openness of the heating system is included as a contextual parameter as it impacts the tap-water system. The hot water obtained from the primary network first goes through the heating system to heat the room, and after that, the water is used by the tap-water system. The valve in the heating system lets out this water and hence can be considered as a context from the tap-water system point of view. It can also be noted that during the non-heating seasons, that is, when the outdoor temperature is above 17 °C, the heating system valve is completely closed and the heat obtained from the primary network is only used to heat the tap-water.

Initial clustering in all the chunks and for all the three views is done using *k*-means clustering, for which SI is used to determine the optimal number of clusters. Table 13 presents details about the number of initial clusters considered for each of the data chunks.

When the global model is updated after the arrival of chunk 2, the concept lattice generated contained 132 non-empty concepts, of which 59 concepts connected all three views. After using the closed patterns, the model has 48 concepts connecting any two views of the local models and 16 connecting all three views. Table 14 represents all these 16 concepts.

As explained earlier, VOM3 is the valve openness mean of a three-way valve used in the tap-water system. It has an opening for letting in the cold water when the temperature of the water is above 60 °C. Opening this valve for letting in cold water is not the desired function, as it leads to energy waste, i.e., the water is initially heated and then cooled down. So, in the desired functionality, the valve of VOM3 should be close to 100, representing that the valve only allows hot water to go through. It can be observed from Table 14 that the model was able to categorize the concepts (10, 13, 14, and 15), where the average values for VOM3 are a lot less than 100, implicating that the valve was opened to let in cold water to maintain the water temperature, which is not desired. This can help the domain experts to analyze the identified situation and detect what went wrong. It is interesting to note that two out of these four (concepts 15 and 14) occurred when hot water consumption was high. All the four concepts occurred during the heating season, that is, when the outdoor temperature is below 15 °C. This reflects the heating system’s impact on the tap-water system as discussed previously when explaining categorizing the features into different views.

The global model is again updated after receiving chunk 3. This time, the generated concept lattice has 63 non-empty concepts, of which 35 concepts link all three views. After the closed patterns are used, there are 35 concepts linking any two views and 18 concepts linking all three views. Table 15 presents all these 18 concepts. Similar to what is observed for the model generated on the first two chunks (Table 14), the average VOM3 values are not close to 100 during the heating season in four concepts (0, 12, 16, and 17). This is explainable as there are influences from the heating system when it is running. It can also be observed in these concepts that the supply temperature ($TW_{IST}$) of the tap-water system is over the natural threshold (55 °C), which is expected. Furthermore, the values for $TW_{FST}$ are high when compared with other values.

#### 5.2.2. Integrated Analysis of Heating and Tap-Water Systems

For the third experimental scenario, data from both heating and tap-water sub-systems, which are part of an HVAC&R system, are considered. Based on the experiments performed on the tap-water system, it is already observed that the tap-water system is influenced by the heating system, especially during the heating season. Therefore, the following experiment is performed to get a deeper insight of into how these systems work in coordination. Along with being able to highlight the correlations between different views of both systems, this experiment also showcases the flexibility of the proposed algorithm. The local models produced during the experiments of the heating and tap-water systems can be directly used to build a new global model representing the relations between all six views (note that the number of views selected could be dynamically changed based on the requirement).

Two global models are built to find the correlations between both systems, one after receiving chunk 2 and the other after receiving chunk 3. In the first iteration, the global model has 892 non-empty concepts, while in the second iteration, the number of concepts is reduced to 382. This could have also been due to fewer instances available when chunks 2 and 3 are combined compared to the combination of chunks 1 and 2.

Table 16 and Table 17 present the concepts retained after using the closed patterns (8, 10 concepts after chunk 2 and 3 have arrived, respectively). These concepts present the correlation between all six views from both the systems. Note that all the features available in the global model are not presented in the tables, as they are too many. Some interesting features that represent the relations between the considered systems are selected.

From Table 16, a deviating behavior can be observed for concept 4. VOM deviates from the patterns seen in other concepts. In addition, it is interesting to see that VOM3 has the greatest drop (6.03%) in value from 100% when compared to other concepts. That is, the valve lets in cold water to reduce the hot tap-water temperature, which is not a desired function. Concept 4 also shows a sudden drop in the trends of the $TW_{V}$. Based on the observed patterns, it is expected to have an increase in hot water usage as the temperature decreases, but this is not the case for this concept.

Concept 0 has a deviating behavior with respect to PHL, i.e., 3 kW (note that this was also identified when analyzing the heating sub-system individually). If one takes a closer look at the features considered for the tap-water system, it can be observed that the ΔP and $TW_{V}$ are the highest during this period. It is interesting to observe this, as having higher ΔP is considered a desired functionality since the system is consuming the energy provided. However, when the raw data are investigated it is noticed that the primary supply and return temperatures are constant at 89 and 38, respectively, leading to a value of 51 for ΔP. This represents a potential fault in these sensors. Based on these for concept 0, it is concluded that there could have been some issues with the sensors collecting the PHL and primary supply and return temperatures data.

For concept 2, it can be observed that the openness of VOM2 (17.43%) is unusually high compared to the other concepts and can be interpreted as a deviating behavior. It is interesting to note that the same concept is retained even after receiving chunk 3 (concept 7 from Table 17), implicating that the data characteristics do not match with any of the new data, which further solidifies that it might be a deviating concept. Interestingly, when the domain expert investigates the system to find the actual cause, it is found to be strange but expected behavior. This concept is easily identified in the integrated scenario compared to the results only from the tap water system as there is more than one concept with similar behavior. This demonstrates that the integrated scenario can help identify trends not visible in individual system analysis. The influence of these systems on each other can reveal hidden patterns and deviating behaviors.

In Table 17, concept 0 has a deviating behavior with respect to both the PHL (0 kW) and ΔP (−19.18 °C). It also has a negative SE value (−420%) which is out of range of the normal SE values; hence it can be concluded that there were issues with the system during this time (this was also identified while analyzing the heating sub-system).

Similar to what was observed in the tap-water system, one can see that the VOM3 has deviated from the desired average value of 100% (concepts 4,7 from Table 16 and concepts 3,6 from Table 17) mainly during the heating seasons and when the outdoor temperature is close to 0 °C. It is interesting to note that the SE and VOM both show acceptable values for this period.

The above analysis shows that the integrated global models built using the sub-systems’ local models can also represent the deviating behaviors observed while analyzing each system individually. This provides the opportunity to have a high-level overview of correlations between both considered systems and helps identify deviating concepts that were not so obvious to identify when only a single system is considered.

## 6. Applicability and Limitations

In this study, we investigate the use of the MV Multi-Instance Clustering approach proposed in [5] for monitoring smart building systems’ sensor data. Two data mining techniques are developed by applying this approach and are studied in this paper. Those can be used for multi-view analysis, mining, and visualization of sensor data to assist domain experts in monitoring and analyzing different systems’ behavior. One of the techniques considers contextual factors in the analysis of system behaviour and performance. The other focuses on dealing with integrated systems, such as those available in the smart building domain. The proposed MV approach additionally allows the domain experts to set the threshold (support) used to identify frequent patterns based on their interests. Such flexibility enables the domain experts to monitor different sub-systems based on various criteria and objectives. The conducted experiments demonstrate that the proposed data mining techniques are capable of identifying deviating behaviors. In general, the presented data analytic tools may be used in other similar applied scenarios relying on static sensor networks for system monitoring.

In addition to the applicability, we identified three limitations in the current study. First, the study mainly focuses on the sub-systems of the HVAC&R system of a specific building. In the future, we plan to explore and evaluate the algorithm’s performance on other systems and different types of buildings. The second limitation concerns the studied contextual conditions. Currently, only two contextual factors, namely outdoor temperature and the effect of the heating system on the tap-water system, are considered. Other complex parameters representing the social behavior of the people living in the building can be included in the model. One such example is dividing a day into parts representing people’s typical daily activities, e.g., morning, afternoon, evening, and night, or including the day category, i.e., weekday or weekend. The third identified limitation is related to the concept drift. As stated in Section 2.2, there are six different types of concept drifts. The current study does not perform explicit experimentation to test the proposed approach’s ability to detect these drift scenarios. Based on the experiments and results obtained, one can conclude that the approach is able to identify frequent deviating behavior groups (based on the user-defined threshold). However, further analysis needs to be performed to determine the algorithm’s performance in identifying different concept drift types.

## 7. Conclusions and Future Work

In this study, we have demonstrated how our multi-view stream clustering algorithm, entitled MV Multi-Instance Clustering algorithm [5], can be used to analyze and monitor different systems present in a smart building environment. The approach considers the multi-source nature of the smart building data and provides individual context-aware and integrated tools of modeling and analyzing the system behavior. We propose various visualization and data mining techniques that can be used at each step of the proposed algorithm. These visualizations facilitate further perception and understanding of the obtained results and can be used by the domain experts in step-by-step analysis of the system behaviour and performance.

Our multi-view stream clustering algorithm perfectly suits the multi-source nature of the data in the smart building domain usually collected from multiple systems. It can be used to analyze these systems due to its flexible character; i.e., it can dynamically select the views used to build the global model to analyze single or multiple systems together as per the need. This flexibility is demonstrated in our work by analyzing the heating and tap-water systems individually and together. The obtained results have shown that our algorithm has the potential to be used in the smart building domain for monitoring and analyzing system behavior and performance. The approach has successfully identified new trends and deviating or non-desired behavioral modes. The built global model has also showcased various correlations between different views considered. The proposed algorithm can facilitate the domain experts in obtaining more profound insights into systems’ performances and at the same time be able to identify and analyze deviating behavior.

Our future plans include exploring other smart building systems and richer contextual conditions. For example, the ventilation sub-system, which is also a part of the HVAC&R system, could be included in the analysis as heating, tap-water, and ventilation sub-systems affect one another. In addition, in order to reduce the effects of social behavior of people on the analysis, we are interested in studying contextual factors. Note that each building has a unique and recurring social behavior patterns and energy usage. Furthermore, the ability of the algorithm in identifying different types of concept drift will be investigated. Finally, we plan to work in the direction of building a user-friendly prototype of the algorithm with the proposed visualizations at each phase so that the domain experts can directly use it in their regular day-to-day analysis of the systems.

## Figures and Tables

**Table 1:** Features used for monitoring the heating system.

View	Id	Feature Name		Acronyms	Units
Operation	1	Secondary Supply Temperature		SST	°C
		Secondary Return Temperature		SRT	°C
		Primary Heat Load		PHL	kW
Performance	2	Valve Openness Mean		VOM	%
		Valve Openness Standard Deviation		VOS	%
		Sub-station Efficiency		SE	%
Context	3	Outdoor Temperature Mean		OTM	°C
		Outdoor Temperature Standard Deviation		OTS	°C

Note. Sub-station Efficiency (SE) is computed as the difference between primary supply temperature (PST) and primary return temperature (PRT) divided by the difference between PST and secondary return temperature (SRT).

**Table 2:** Initial number of clusters in three chunks for each view—Heating System.

Chunk	View 1	View 2	View 3
1	5	3	4
2	7	3	4
3	4	3	4

**Table 3:** Operating modes identified after receiving chunk 2.

Operating Modes (OM)	PHL (kW)	SST (°C)	SRT (°C)	Size
$A_{O0}$	24.16	43.88	38.03	118
$A_{O1}$	12.27	34.48	32.23	80
$A_{O2}$	4.83	28.41	26.78	160
$A_{O3}$	44.38	49.52	39.55	49
$A_{O4}$	3.33	42.51	36.16	34
$A_{O5}$	19.07	45.28	39.47	12
$A_{O6}$	18.29	39.68	36.12	18
$A_{O7}$	29.29	47.46	39.63	16
$A_{O8}$	10.35	31.11	29.68	26

**Table 4:** Performance modes identified after receiving chunk 2.

Performance Modes (PM)	SE (%)	VOM (%)	VOS (%)	Size
$A_{P0}$	96	14.04	±1.16	338
$A_{P1}$	70	0.95	±0.08	45
$A_{P2}$	75	7.46	±4.81	98
$A_{P3}$	76	6.97	±4.43	32

**Table 5:** Contextual conditions identified after receiving chunk 2.

Contextual Conditions (CC)	OTM (°C)	OTS (°C)	Size
$A_{C0}$	2.57	±1.18	131
$A_{C1}$	6.53	±1.97	168
$A_{C2}$	19.41	±2.91	122
$A_{C3}$	13.20	±2.39	61
$A_{C4}$	11.73	±3.27	31

**Table 6:** Patterns representing the behavior of heating system between 1 to 7 March 2019 and 15 to 21 March 2019.

Date	OM	PM	CC	Date	OM	PM	CC
1 March 2019	$A_{O3}$	$A_{P0}$	$A_{C1}$	15 March 2019	$A_{O4}$	$A_{P0}$	$A_{C1}$
2 March 2019	$A_{O0}$	$A_{P0}$	$A_{C1}$	16 March 2019	$A_{O4}$	$A_{P0}$	$A_{C1}$
3 March 2019	$A_{O0}$	$A_{P0}$	$A_{C1}$	17 March 2019	$A_{O4}$	$A_{P0}$	$A_{C1}$
4 March 2019	$A_{O3}$	$A_{P0}$	$A_{C1}$	18 March 2019	$A_{O4}$	$A_{P0}$	$A_{C1}$
5 March 2019	$A_{O3}$	$A_{P0}$	$A_{C1}$	19 March 2019	$A_{O4}$	$A_{P0}$	$A_{C1}$
6 March 2019	$A_{O3}$	$A_{P0}$	$A_{C1}$	20 March 2019	$A_{O4}$	$A_{P0}$	$A_{C1}$
7 March 2019	$A_{O0}$	$A_{P0}$	$A_{C1}$	21 March 2019	$A_{O4}$	$A_{P0}$	$A_{C1}$

**Table 7:** Closed patterns from global model showing correlations between all three views after receiving chunk 2.

S/N	PHL	SST	SRT	VOM	VOS	SE	OTM	OTS	Size	Months
6	3.37	26.85	26.60	0.00	± 0.00	70	22.12	± 3.01	40	[6–8]
10	4.99	29.14	26.68	6.53	± 4.96	71	18.23	± 2.59	62	[6–8]
1	4.75	26.59	26.25	5.60	± 4.90	78	17.88	± 3.79	19	[6]
2	6.6	29.75	27.02	8.47	± 4.61	78	16.68	± 2.19	21	[5, 9]
3	12.95	33.73	32.09	11.65	± 1.30	88	11.12	± 2.55	27	[5, 9]
5	12.9	35.28	32.87	11.87	± 1.04	92	9.68	± 1.96	34	[ 3, 4, 10, 11]
4	3.33	42.51	36.16	15.53	± 1.55	96	5.47	± 2.01	34	[ 3, 4, 11]
9	24.68	44.00	37.95	14.21	± 0.99	99	4.41	± 1.05	60	[ 1, 2, 12]
8	23.61	43.93	38.26	13.78	± 0.88	97	4.40	± 1.33	54	[ 3, 4, 10, 11]
0	29.62	47.69	39.75	15.16	± 1.23	104	1.02	± 1.85	14	[1, 2]
7	45.02	49.58	39.54	18.28	± 1.61	100	−0.59	± 1.20	42	[ 1, 2, 12]

Note. The unit for PHL is kW and for SST, SRT, OTM, and OTS is °C. VOM, VOS, and SE are expressed in %. For the full form of each feature, see Table 1.

**Table 8:** Operating modes identified after receiving chunk 3.

Operating Modes (OM)	PHL (kW)	SST (°C)	SRT (°C)	Size
$B_{O0}$	21.17	42.22	36.73	59
$B_{O1}$	4.03	26.69	25.57	137
$B_{O2}$	19.07	45.28	39.47	12
$B_{O3}$	29.29	47.46	39.63	16
$B_{O4}$	13.45	34.78	32.54	17
$B_{O5}$	10.35	31.11	29.68	26
$B_{O6}$	0.70	33.99	30.02	33
$B_{O7}$	0.00	44.00	35.98	29

**Table 9:** Performance modes identified after receiving chunk 3.

Performance Modes (PM)	SE (%)	VOM (%)	VOS (%)	Size
$B_{P0}$	−23	9.12	±1.33	308
$B_{P1}$	−2785	13.61	±1.23	10
$B_{P2}$	2894	14.14	±1.32	11

**Table 10:** Contextual conditions identified after receiving chunk 3.

Contextual Conditions (CC)	OTM (°C)	OTS (°C)	Size
$B_{C0}$	3.99	±1.07	75
$B_{C1}$	19.61	±3.02	86
$B_{C2}$	6.93	±2.52	46
$B_{C3}$	11.73	±3.27	31
$B_{C4}$	14.65	±1.98	30
$B_{C5}$	8.58	±1.20	61

**Table 11:** Closed patterns from global model showing correlations between all three views after receiving chunk 3.

S/N	PHL	SST	SRT	VOM	VOS	SE	OTM	OTS	Size	Months
13	4.25	26.58	25.67	1.78	±1.27	74	19.76	±3.02	80	[6–8]
1	6.89	28.16	26.73	8.30	±4.32	75	15.47	±3.66	8	[5]
11	5.23	25.99	24.66	9.77	±2.89	21	14.65	±1.98	30	[9]
9	0.00	27.63	26.11	11.16	±0.50	−293	12.38	±0.90	19	[10, 11]
7	10.23	31.46	29.67	10.63	±1.66	81	11.36	±3.26	15	[5]
5	10.52	30.62	29.69	11.05	±1.20	90	11.19	±3.16	11	[4]
0	13.81	35.43	32.29	11.17	±1.18	89	8.69	±2.89	8	[5]
10	0.00	33.82	30.68	12.09	±0.95	−506	8.46	±1.38	22	[10, 11]
2	13.13	34.20	32.77	11.47	±1.16	90	8.0	±3.00	9	[4]
8	21.05	41.07	36.72	13.17	±0.93	100	5.59	±2.2	18	[3, 4]
3	0.00	41.43	34.89	13.45	±1.16	−787	5.27	±1.71	10	[10, 11]
12	21.22	42.73	36.73	13.22	±0.99	99	5.13	±0.96	41	[ 1, 2, 12]
4	0.0	44.98	36.76	14.14	±1.33	2852	3.75	±0.58	10	[12]
6	29.62	47.69	39.75	15.16	±1.23	104	1.02	±1.85	14	[1, 2]

Note. The unit for PHL is kW and for SST, SRT, OTM, and OTS is °C. VOM, VOS, and SE are expressed in %. For the full form of each feature see Table 1.

**Table 12:** Features used for monitoring the tap-water system.

View	Id	Feature Name		Acronyms	Units
Operation	4	Tap-water Initial Supply Temperature		$TW_{IST}$	°C
		Tap-water Final Supply Temperature		$TW_{FST}$	°C
		Tap-water Return Temperature		$TW_{RT}$	°C
		Primary Heat Load		PHL	kW
		Tap-water Volume Consumed		$TW_{V}$	m${}^{3}$
Performance	5	Tap-water Valve Openness Mean 2		VOM2	%
		Tap-water Valve Openness Standard Deviation 2		VOS2	%
		Tap-water Valve Openness Mean 3		VOM3	%
		Primary Delta		ΔP	°C
Context	6	Outdoor Temperature Mean		OTM	°C
		Valve Openness Mean (Heating system)		VOM	%

**Table 13:** Initial number of clusters in three chunks for each view—Tap-water System.

Chunk	View 4	View 5	View 6
1	6	5	5
2	7	5	4
3	3	6	7

**Table 14:** Closed patterns from global model showing correlations between all three views after receiving chunk 2. Values are sorted based on OTM followed by $TW_{V}$.

S/N	PHL	${\mathrm{TW}}_{\mathrm{IST}}$	${\mathrm{TW}}_{\mathrm{FST}}$	${\mathrm{TW}}_{\mathrm{RT}}$	${\mathrm{TW}}_{V}$	VOM2	VOS2	VOM3	ΔP	OTM	VOM	Size	Months
4	2.58	57.09	56.33	53.97	0.30	5.06	±10.37	98.51	33.26	22.81	0.00	16	[6–9]
8	5.21	56.93	56.24	53.80	0.30	5.18	±11.30	99.01	35.64	18.15	7.31	24	[5–8]
2	5.22	56.7	55.94	53.50	0.68	8.62	±13.77	98.77	39.54	18.17	7.17	15	[5–9]
12	5.55	55.55	54.87	52.47	0.55	8.82	±12.33	99.85	34.08	17.47	7.44	28	[5–9]
3	5.04	55.02	54.38	51.96	0.62	17.07	±9.18	99.99	38.77	16.95	6.22	15	[5, 6]
7	11.56	55.72	55.05	52.68	0.71	10.49	±13.41	99.86	38.83	11.94	11.32	22	[ 4–6, 9, 10]
1	9.38	57.06	56.51	53.85	0.24	6.02	±12.02	98.24	41.14	11.60	11.27	15	[1, 4, 5, 7, 9]
9	13.60	56.57	55.87	53.43	0.84	8.92	±13.35	99.31	40.17	10.70	11.60	25	[ 4, 5, 7, 9, 10]
10	11.52	57.08	56.20	53.50	0.85	11.22	±14.21	93.58	51.20	10.26	11.44	26	[2–5]
11	26.48	56.04	55.75	52.67	0.95	11.13	±13.91	99.62	39.40	5.18	15.02	28	[ 1–3, 5, 10, 12]
0	16.05	56.98	56.30	53.77	0.24	6.33	±10.97	98.36	43.71	4.99	13.72	13	[ 1–4, 10–12]
6	21.86	56.97	56.12	53.68	0.85	7.59	±11.95	98.58	40.24	4.71	13.60	17	[1–3, 10–12]
15	14.14	56.80	56.23	53.32	0.95	11.48	±15.02	96.45	49.05	4.48	14.20	30	[ 2–4 11, 12]
13	22.04	58.9	57.60	54.92	0.60	4.60	±10.18	94.78	43.8	3.87	13.11	29	[1–3, 5, 10–12]
5	37.24	56.43	56.11	52.73	0.81	9.35	±14.07	99.47	38.38	2.09	17.43	17	[ 1–3, 12]
14	46.81	58.64	57.57	53.92	1.00	5.84	±11.87	95.83	42.53	−1.66	18.42	30	[ 1–3, 12]

Note. The units for PHL and $TW_{V}$ are kw and m${}^{3}$, respectively. The unit for $TW_{IST}$, $TW_{FST}$, $TW_{RT}$, ΔP, and OTM is °C. VOM, VOM2, VOS2, and VOM3 are expressed in %. For the full form of each feature see Table 12.

**Table 15:** Closed patterns from global model showing correlations between all three views after receiving chunk 3. Values are sorted based on OTM followed by $TW_{V}$.

S/N	PHL	${\mathrm{TW}}_{\mathrm{IST}}$	${\mathrm{TW}}_{\mathrm{FST}}$	${\mathrm{TW}}_{\mathrm{RT}}$	${\mathrm{TW}}_{V}$	VOM2	VOS2	VOM3	ΔP	OTM	VOM	Size	Months
6	3.34	55.21	54.66	52.31	0.48	6.97	±8.52	100.00	32.55	23.03	0.00	11	[6–8]
15	3.99	55.34	54.55	52.20	0.56	16.39	±10.71	100.00	34.21	22.33	0.26	23	[6–8]
14	4.30	54.90	54.24	51.77	0.64	17.10	±9.73	100.00	35.87	18.24	0.01	20	[7, 8]
7	4.00	54.96	54.28	51.98	0.59	10.39	±10.65	100.00	38.30	17.99	0.00	11	[7, 8]
11	5.04	55.02	54.38	51.96	0.62	17.07	±9.18	99.99	38.77	16.95	6.22	15	[5, 6]
8	7.14	54.85	54.44	51.62	0.72	9.10	±9.02	100.00	50.96	14.63	10.31	11	[9]
4	0.00	54.88	54.49	51.58	0.65	16.14	±9.03	100.00	−13.48	14.25	10.61	10	[ 9, 10]
13	0.00	54.84	54.49	51.64	0.77	12.10	±10.35	100.00	−21.36	9.88	11.53	16	[10, 11]
16	13.75	56.78	55.88	53.35	0.74	10.89	±13.18	94.62	49.60	9.27	11.59	33	[1, 2, 4, 5, 9]
12	13.11	59.49	57.53	54.81	0.57	7.01	±12.30	90.68	47.41	9.03	11.35	16	[2, 4, 5]
1	0.00	54.94	54.57	51.63	0.61	7.05	±7.30	100.00	−17.17	8.92	11.52	8	[10, 11]
2	0.00	54.85	54.45	51.69	0.90	11.76	±10.99	100.00	−22.76	6.94	13.35	9	[11, 12]
9	0.00	54.90	54.42	51.67	0.57	7.09	±8.73	100.00	−18.08	5.97	13.04	12	[10–12]
5	20.65	54.93	54.50	51.68	0.64	9.62	±9.65	100.00	47.74	5.48	12.97	10	[12]
0	20.27	58.80	57.93	55.14	0.70	5.56	±12.97	93.90	46.09	3.97	12.98	8	[1–3]
10	25.04	55.58	54.78	52.39	0.99	15.93	±12.43	99.12	42.64	3.95	14.60	13	[1–4]
3	0.00	54.85	54.42	51.68	0.82	10.45	±10.84	100.00	−21.17	3.34	14.15	9	[11, 12]
17	24.33	56.37	55.48	53.18	0.79	10.62	±12.41	96.69	45.29	2.77	13.97	38	[ 1–4, 12]

Note. The units for PHL and $TW_{V}$ are kw and m${}^{3}$, respectively. The unit for $TW_{IST}$, $TW_{FST}$, $TW_{RT}$, ΔP, and OTM is °C. VOM, VOM2, VOS2, and VOM3 are expressed in %. For the full form of each feature see Table 12.

**Table 16:** Closed patterns from global model showing correlations between all six views after receiving chunk 2. Values are sorted based on OTM.

S/N	PHL	SST	SSR	VOM	SE	OTM	${\mathrm{TW}}_{\mathrm{IST}}$	${\mathrm{TW}}_{\mathrm{FST}}$	${\mathrm{TW}}_{\mathrm{RT}}$	${\mathrm{TW}}_{V}$	VOM2	VOM3	ΔP	Size	Months
1	2.66	27.02	26.53	0.00	67	23.15	56.96	56.29	53.95	0.29	5.36	98.50	33.24	14	[6–8]
6	4.83	29.51	26.83	7.23	70	18.19	56.89	56.26	53.74	0.30	5.03	99.16	35.51	21	[7, 8]
3	5.40	29.38	26.86	7.25	69	17.95	55.69	55.00	52.64	0.45	7.71	99.72	31.52	15	[6–8]
2	5.03	26.57	26.08	6.16	80	16.92	55.01	54.36	51.94	0.61	17.43	100.00	39.14	14	[6]
0	3.00	42.12	36.19	14.83	97	5.60	56.49	56.47	52.95	0.97	11.79	98.57	51.00	14	[3, 4]
5	26.56	43.09	36.82	15.09	100	5.23	55.88	55.73	52.53	0.95	11.34	99.72	39.07	19	[1, 2, 12]
4	22.52	44.61	39.25	13.12	96	3.85	58.85	57.47	54.84	0.69	4.57	93.97	43.93	16	[10, 11]
7	47.60	50.89	40.04	18.58	99	−1.70	58.64	57.58	53.90	1.00	5.78	95.62	42.42	27	[1, 2]

Note. The units for PHL and $TW_{V}$ are kw and m${}^{3}$, respectively. The unit for $TW_{IST}$, $TW_{FST}$, $TW_{RT}$, ΔP, and OTM is °C. VOM, VOM2, and VOM3 are expressed in %. For the full form of each feature see Table 1 and Table 12.

**Table 17:** Closed patterns from global model showing correlations between all six views after receiving chunk 3. Values are sorted based on OTM.

S/N	PHL	SST	SSR	VOM	SE	OTM	${\mathrm{TW}}_{\mathrm{IST}}$	${\mathrm{TW}}_{\mathrm{FST}}$	${\mathrm{TW}}_{\mathrm{RT}}$	${\mathrm{TW}}_{V}$	VOM2	VOM3	ΔP	Size	Months
4	3.34	27.20	26.10	0.00	70	23.03	55.21	54.66	52.31	0.48	6.97	100.00	32.55	11	[6–8]
9	4.05	26.47	26.14	0.27	74	22.41	55.36	54.56	52.21	0.56	16.45	100.00	34.56	22	[6–8]
1	3.92	26.72	24.94	0.00	75	18.08	54.92	54.24	51.94	0.57	10.33	100.00	38.39	10	[7, 8]
8	4.39	26.32	24.83	0.01	74	18.53	54.91	54.25	51.77	0.65	16.46	100.00	36.39	16	[7, 8]
7	5.03	26.57	26.08	6.16	80	16.92	55.01	54.36	51.94	0.61	17.43	100.00	39.14	14	[6]
5	7.14	25.82	24.26	10.31	83	14.63	54.85	54.44	51.62	0.72	9.10	100.00	50.96	11	[9]
0	0.00	32.00	29.86	11.71	−420	9.10	54.83	54.46	51.6	0.75	11.81	100.00	−19.18	8	[10, 11]
2	20.65	42.07	35.52	12.97	97	5.48	54.93	54.50	51.68	0.64	9.62	100.00	47.74	10	[12]
6	24.32	44.84	37.66	13.79	101	3.40	55.92	55.15	52.70	0.73	9.80	97.95	46.02	12	[1, 2, 12]
3	29.51	47.75	39.80	15.10	104	0.87	56.48	55.47	53.14	1.03	12.71	95.69	46.33	11	[1, 2]

Note. The units for PHL and $TW_{V}$ are kw and m${}^{3}$, respectively. The unit for $TW_{IST}$, $TW_{FST}$, $TW_{RT}$, ΔP, and OTM is °C. VOM, VOM2, and VOM3 are expressed in %. For the full form of each feature see Table 1 and Table 12.

## Abbreviations

The following abbreviations are used in this manuscript:

CC	Contextual Conditions
DH	District Heating
HVAC&R	Heating, Ventilation, Air Conditioning and Refrigeration
MAD	Median Absolute Deviation
MI	Multi-Instance
ML	Machine Learning
MV	Multi-View
MV-MIC	MV Multi-Instance Clustering
OM	Operating Modes
OTM	Outdoor Temperature Mean
OTS	Outdoor Temperature Standard Deviation
PM	Performance Modes
PHL	Primary Heat Load
SRT	Secondary Return Temperature
SST	Secondary Supply Temperature
SI	Silhouette Index
SE	Sub-station Efficiency
$TW_{FST}$	Tap-water Final Supply Temperature
$TW_{IST}$	Tap-water Initial Supply Temperature
$TW_{RT}$	Tap-water Return Temperature
$TW_{V}$	Tap-water Volume Consumed
VOM	Valve Openness Mean
VOS	Valve Openness Standard Deviation
